# Diagnostic accuracy of PSMA-targeted radioguided surgery in prostate cancer at multiple anatomical levels: a systematic review and meta-analysis

**DOI:** 10.1007/s00259-026-07773-x

**Published:** 2026-03-27

**Authors:** Fang Wen, Laura Schäfer, Xinlin Zheng, Hao Huang, Walter Noordzij, Matthias Saar, Felix M. Mottaghy, Susanne Lütje

**Affiliations:** 1 Department of Nuclear Medicine, University Hospital RWTH Aachen, Aachen, Germany; 2 Department of Nuclear Medicine and Molecular Imaging, University Medical Center Groningen, University of Groningen, Groningen, The Netherlands; 3 Department of Urology and Pediatric Urology, University Hospital RWTH Aachen, Aachen, Germany; 4 Center for Integrated Oncology (CIO), University Hospital RWTH Aachen, Aachen, Germany; 5 Department of Radiology and Nuclear Medicine, Maastricht University Medical Center, Maastricht, The Netherlands

**Keywords:** PSMA-RGS, Radioguidance, Intraoperative detection, Prostate cancer, Lymph node dissection

## Abstract

**Background:**

Prostate-specific membrane antigen (PSMA)-targeted radioguided surgery (PSMA-RGS) is a promising intraoperative technique for improving lesion localization and surgical accuracy in prostate cancer (PCa), particularly in high-risk or recurrent cases.

**Objective:**

To systematically evaluate the diagnostic performance of PSMA-RGS in PCa using multilevel meta-analysis.

**Methods:**

Following PRISMA 2020 guidelines, we searched PubMed, Embase, and Web of Science for clinical studies published between January 2016 and May 2025, with the final search performed on May 1, 2025, and identified 28 eligible studies. Diagnostic accuracy was assessed at the patient, lesion, region, and lymph node levels, using pooled sensitivity, specificity, likelihood ratios, and diagnostic odds ratios (DOR). Random-effects meta-analyses were conducted and risk of bias was evaluated with the ROBINS-I tool.

**Results:**

Ex vivo PSMA-RGS demonstrated the highest specificity (up to 100%), while in vivo PSMA-RGS showed excellent sensitivity (90–97%) and specificity (90–99%), particularly at the lymph node level. Across all evaluated anatomical levels, PSMA-RGS consistently outperformed preoperative imaging (log DOR: 5.77 vs. 2.99; *p* < 0.0001). All diagnostic results were confirmed by histopathology as the reference standard. Meta-regression identified in vivo PSMA-RGS, lymph node–based analysis, and γ-probes combined with high-purity germanium detectors—used for post-resection confirmation—as independent predictors of improved diagnostic performance.

**Conclusion:**

PSMA-RGS demonstrates robust diagnostic accuracy across multiple analytical levels, including per-patient, per-lesion, and per-node assessments. Initial evidence suggests that intraoperative γ-probes guidance may enable real-time localization of otherwise undetected nodal metastases. In high-risk prostate cancer surgery, this approach may help refine intraoperative decision-making, improve resection completeness, and reduce recurrence.

**Supplementary Information:**

The online version contains supplementary material available at 10.1007/s00259-026-07773-x.

## Introduction

Prostate cancer (PCa) remains one of the most commonly diagnosed solid malignancies among men worldwide, with over 1.4 million new cases and approximately 375,000 deaths annually [1]. For patients with localized or locally advanced disease, the 2024 EAU–EANM–ESTRO–ESUR–ISUP–SIOG guidelines recommend curative options such as radical prostatectomy (RP), pelvic lymph node dissection (PLND), and external beam radiotherapy (EBRT).These treatments are tailored to individual risk factors [2]. Despite the curative intent of radical prostatectomy, up to 30% of patients experience biochemical recurrence (BCR) within five years [3], often due to positive surgical margins or undetected nodal metastases [4, 5].

In PLND, accurate intraoperative lesion localization—particularly of metastatic lymph nodes—remains a major challenge. Surgical exploration relies heavily on preoperative imaging and anatomical experience; yet, even skilled surgeons may miss micrometastatic or fibrotic nodes, especially during robot-assisted procedures where tactile feedback is absent [6, 7]. Conventional imaging modalities such as computed tomography (CT) or magnetic resonance imaging lack sufficient sensitivity for sub-5 mm lesions. Prostate-specific membrane antigen (PSMA)-targeted positron emission tomography/computed tomography (PET/CT) has emerged as the most sensitive tool for staging and detecting PCa. However, even when suspicious lesions are clearly visualized preoperatively, surgeons often encounter challenges in accurately localizing and resecting them intraoperatively due to the limited translation of imaging information into the surgical field. This discrepancy may lead to incomplete lesion removal despite precise preoperative mapping [8].

Radioguided surgery (RGS) offers a promising solution by combining preoperative molecular imaging with intraoperative γ-probe-based lesion detection. Among available tracers, PSMA-targeted agents stand out due to their high tumor specificity and broad clinical adoption. PSMA-targeted radioguided surgery (PSMA-RGS) enables real-time localization and removal of lesions that are otherwise difficult to identify intraoperatively, even if visible on PET/CT [9–11]. Gamma probes provide immediate feedback on radiotracer uptake, supporting both in vivo lesion localization and ex vivo margin assessment [12]. PSMA-RGS has shown strong diagnostic performance in detecting subcentimeter metastases, even in anatomically challenging regions. For example, the use of [^111^In]In-PSMA-617 achieved a sensitivity and specificity of 92% and 98%, respectively, for differentiating malignant from benign lymph nodes on a per-node basis [13], while Perera et al. confirmed the overall accuracy of PSMA imaging in advanced disease [11]. In multicenter trials, PSMA-RGS improved lesion detection rates during salvage lymphadenectomy and correlated well with histopathology findings [10, 14]. Intraoperative specimen PET/CT further improved assessment of margins and residual disease, showing strong concordance with histopathology [15]. In recognition of its clinical value, PSMA PET/CT is now endorsed by EAU-EANM consensus guidelines for both staging and theranostic applications, including [^1^⁷⁷Lu]Lu-PSMA therapy [16]. Although not yet incorporated into current guidelines, PSMA-RGS is emerging as a promising intraoperative adjunct, with accumulating evidence supporting its potential to enhance lesion detection and surgical completeness.

Emerging evidence also suggests a potential oncological benefit of PSMA-RGS. In selected patients with oligometastatic disease or low preoperative PSA levels, PSMA-RGS has been associated with complete biochemical response rates of up to 66% and recurrence-free survival exceeding 19 months [17, 18].

This systematic review aims to analyze the current evidence regarding the efficacy, safety, and clinical utility of PSMA-RGS in PCa. Although various radiotracers, such as choline- or GRPR-targeted agents, have been explored, we focus on PSMA-targeted approaches due to their superior tumor specificity, clinical adoption, and stronger evidence base [11, 19]. By analyzing the performance, surgical margin outcomes, and survival endpoints, we seek to identify gaps in the existing literature and provide direction for future clinical research and standardization efforts.

## Evidence acquisition

### Search strategy

This study followed the PRISMA 2020 statement and Cochrane Handbook guidelines to conduct a systematic literature search [20]. The review protocol was prospectively registered in the International Prospective Register of Systematic Reviews (PROSPERO; registration ID: CRD420251115458).A systematic search was conducted in PubMed, Embase, and Web of Science for studies published from January 1, 2016, to May 1, 2025. The final search update was performed on May 1, 2025, before manuscript submission.The search combined MeSH/Emtree terms and free-text keywords, focusing on three core concept modules: (1) prostate cancer (PCa); (2) surgical procedures (e.g., prostatectomy, lymphadenectomy); and (3) intraoperative imaging or radiotracers (e.g., radioguided surgery, PSMA-targeted imaging, PET/CT, gamma probe, fluorescence). The full electronic search strategies for PubMed, Embase, and Web of Science are provided in Supplementary Tables S1–S3. All included studies were publicly available at the time of the final search.

### Selection criteria

Two authors independently screened all records, and discrepancies were resolved by consensus. First, the following types of studies were excluded: reviews, case reports, conference abstracts, letters, non-English articles, and studies lacking clinical data. Titles and abstracts were screened to exclude irrelevant studies. Full texts were reviewed for eligibility based on predefined criteria. Only clinical trials and observational studies with PSMA-targeted radioguided surgery were included; animal studies and those without clinical data were excluded. Eligible studies were clinical trials or observational cohorts evaluating PSMA-targeted radiopharmaceuticals (e.g.,[^68^ Ga]Ga-PSMA-11, [^99m^Tc]Tc-PSMA-I&S, [^18^F]PSMA-1007). Studies using non-PSMA tracers or animal models were excluded.

### Quality assessment

A total of 28 studies were ultimately included in this study, all of which were systematically assessed for risk of bias using the Risk Of Bias In Non-randomized Studies of Interventions (ROBINS-I) tool. The scoring covered seven dimensions, including control of confounding factors, selection of study subjects, definition of intervention groups, deviation in intervention implementation, data missing, outcome measurement, and completeness of outcome reporting.

### Data extraction

From each study, we extracted: basic characteristics (sample size, age, tumor stage); imaging/surgical parameters (e.g., PET/CT type, open or robot-assisted surgery, tracer type); clinical context (initial vs. recurrent setting); prior treatments; preoperative PSA; pathological outcomes (e.g., positive surgical margin (PSM), node positivity); follow-up endpoints (e.g., biochemical recurrence, response); and safety outcomes (e.g., surgical complications). For diagnostic studies, true positive (TP), false positive (FP), true negative (TN), and false negative (FN) values were retrieved to calculate diagnostic accuracy. Data were stratified where needed to preserve subgroup integrity. For this review, in vivo RGS was defined as intraoperative γ-probe detection of lesions in the patient body during surgery, whereas ex vivo RGS referred to radioguided assessment of resected specimens to confirm residual disease. Data extraction was performed using a standardized Excel form by two independent reviewers.

### Statistical analysis

Meta-analysis was conducted in R (version 4.3.2) using packages meta, metafor, mada, and ggplot2. Diagnostic metrics (sensitivity, specificity, PLR, NLR, DOR) were pooled via a random-effects model (DerSimonian–Laird) with logit transformation. Forest plots were generated with 95% confidence intervals (CIs).

Heterogeneity was assessed using Cochran's Q test for homogeneity and the I^2^ statistic for heterogeneity, with I^2^ > 50% or *p-*value < 0.10 indicating significant heterogeneity. Sensitivity analysis was conducted via the leave-one-out method. Publication bias was assessed by funnel plots. Given the limited number of studies, the trim-and-fill method was applied for adjustment. A Fagan nomogram (pre-test probability set at 20%) and a log-PLR vs. log-NLR plot were constructed for clinical interpretation. All statistical tests were two-sided, with* p* < 0.05 considered statistically significant. For subgroup analysis of intraoperative probes, studies that reported γ-probes in combination with high-purity germanium detectors were analyzed separately, as these detectors—despite being used postoperatively—were integral to the radioguided workflow and may influence diagnostic discrimination.

## Results

### Study selection

**Fig. 1:**
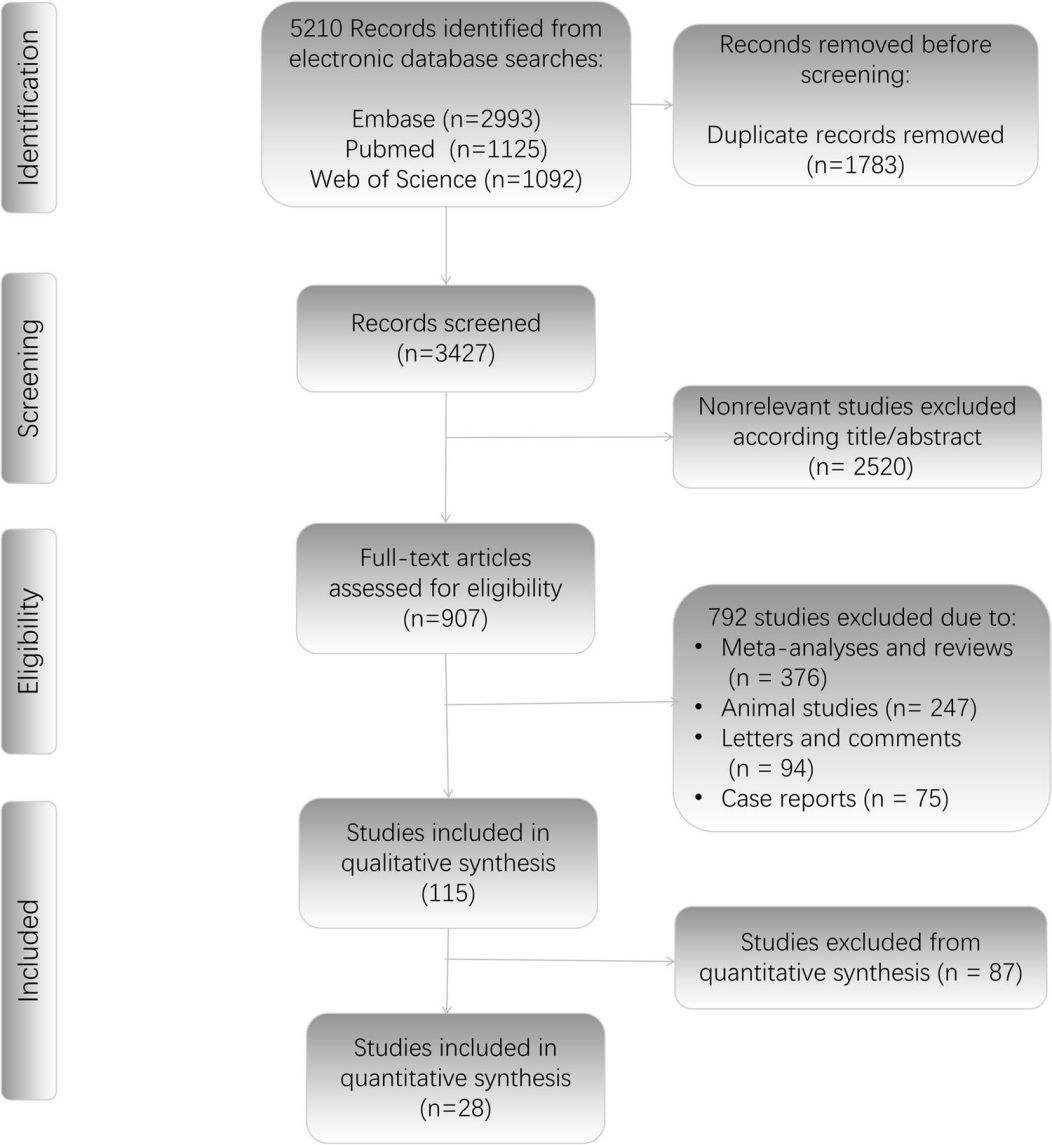
PRISMA flow diagram of the study selection process

A total of 5210 records were identified from PubMed (*n* = 1125), Embase (*n* = 2993), and Web of Science (*n* = 1092). After removal of 1783 duplicates, 3427 records underwent title and abstract screening. Of these, 2520 were excluded. The remaining 907 full-text articles were assessed for eligibility, and 792 were excluded (including reviews, animal studies, case reports, and editorials). Finally, 115 full-text articles were assessed. Ultimately, 28 studies met the inclusion criteria and were included in the final review (Fig. 1). The full electronic search strategies are provided in Supplementary Tables S1–S3.

### Risk of bias assessment

#### Domain-specific risk

Risk of bias was assessed using the ROBINS-I tool across seven domains (see Supplementary Figure S1). Most studies showed low risk in intervention classification, protocol adherence, and outcome measurement. However, high risk of confounding was identified in 32% of studies due to inadequate adjustment for variables such as preoperative PSA, tumor burden, and surgical approach. Participant selection bias was also common due to retrospective designs and insufficiently reported inclusion criteria.

#### Overall bias distribution and methodological quality

**Fig. 2:**
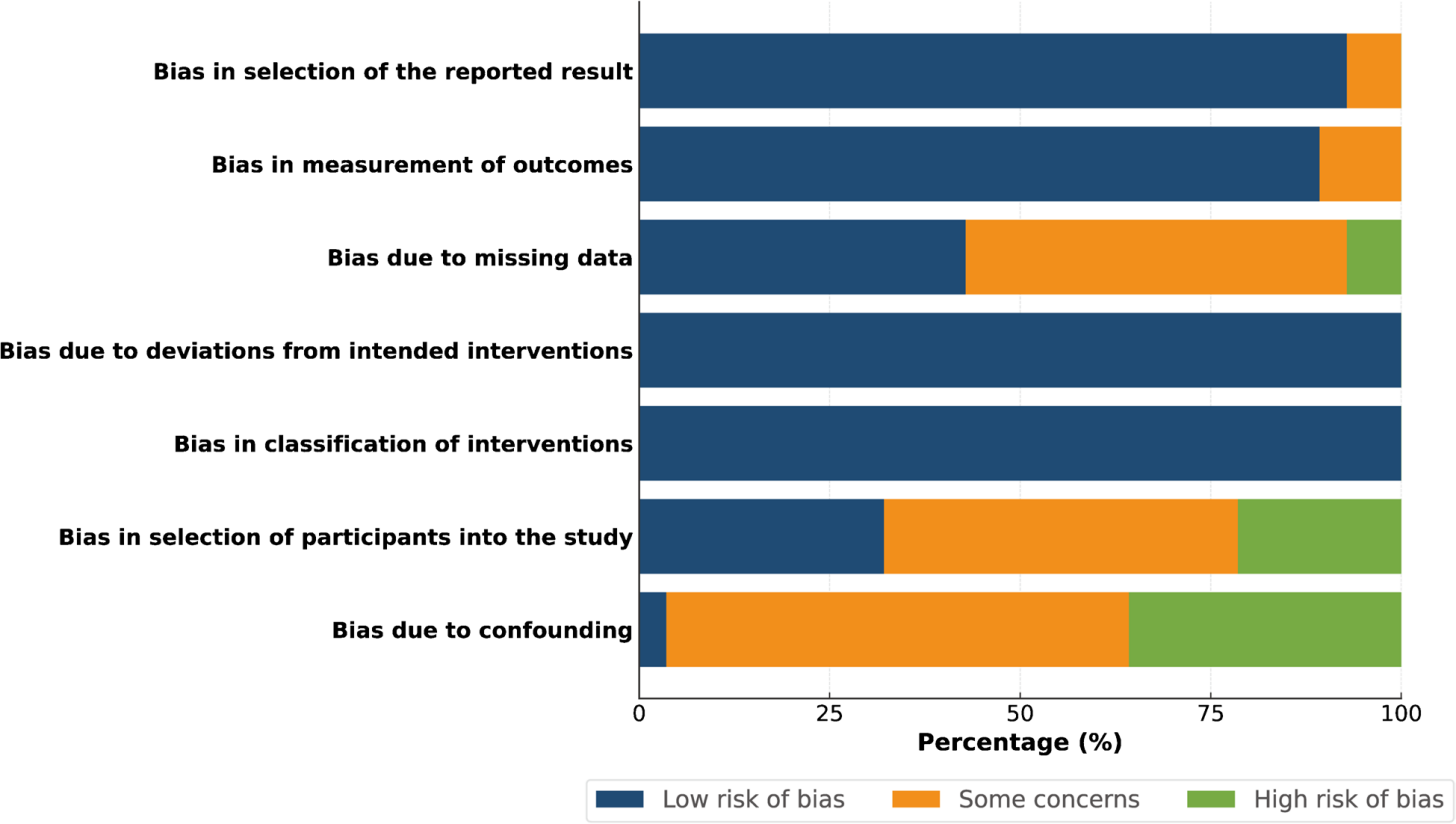
Overall distribution of risk of bias across ROBINS-I domains for all included studies. Proportion of studies classified as low risk, some concerns, or high risk across each of the seven domains

The overall risk of bias is summarized in Fig. 2. While reporting quality was generally acceptable, the presence of methodological heterogeneity and residual confounding highlights the need for future prospective, standardized studies.

### Study characteristics

#### Study and patient characteristics

A total of 28 studies were included in the qualitative synthesis (Table 1). The majority were prospective cohorts (*n* = 19), with the remainder being retrospective cohorts (*n* = 9).Geographically, the studies originated primarily from European centers (Germany, the Netherlands, Italy). The enrolled populations consisted of either patients with high-risk primary prostate cancer or those with biochemical recurrence. Key demographic and methodological characteristics are summarized in Table 1, whereas detailed information on radiotracers, surgical approaches, and intraoperative detection techniques is provided in Supplementary Tables S4 and S5. Among the included studies, [^68^ Ga]Ga-PSMA-11 and [^99m^Tc]Tc-PSMA-I&S were the most frequently used tracers, whereas [^18^F]PSMA-1007 appeared in a minority of reports. Surgical approaches varied between open and robot-assisted procedures, and most cohorts enrolled patients with high-risk or recurrent disease. For age was extracted as reported in the original studies (mean ± SD, median [IQR], median [range], or individual values) without transformation.

**Table 1:** Characteristics of Included Studies (study and patient characteristics; detailed surgical and tracer data are provided in Supplementary Tables S4–S5)

Ref	Author, year	Age (years)	Country	Study Design	Patient Type	Sample Size (*n*)
1 [21]	Collamati, 2020	71,57,73,66,63,55,48^c^	Netherlands/Italy	Prospective cohort	Primary high-risk	7
2 [22]	Jilg, 2020	67.5 ± 6.6 ^a^	Germany	Retrospective cohort	Recurrent Disease or primary cN +	23
3 [23]	Mix,2021	61.0 ± 8.0 ^a^	Germany	Prospective cohort	Recurrent Disease or primary cN +	6
4 [14]	de Barros, 2022	Median 68 (IQR 66–72)^b^	Netherlands	Prospective cohort	Recurrent disease	20
5 [24]	Gondoputro, 2022	Median 68 (IQR 57–69) ^b^	Australia	Prospective cohort	Primary high-risk	12
6 [25]	Knipper, 2023	67 (IQR: 62–71) ^b^	Germany	Retrospective cohort	Recurrent disease	364
7 [26]	Yılmaz, 2022	63.3 ± 6.2 ^a^	Turkey	Prospective cohort	Primary intermediate -/high-risk	15
8 [27]	Gandaglia,2022	70 (IQR: 66–71) ^b^	Italy	Prospective cohort	Primary intermediate -/high-risk	12
9 [28]	Koehler, 2023	Median 62 (IQR 61–67) ^b^	Germany	Retrospective cohort	Recurrent disease	9
10 [29]	Stibbe, 2023	69 (IQR: 64–70) ^b^	Netherlands	Prospective cohort	Primary intermediate -/high-risk	18
11 [30]	Falkenbach, 2025	67 (IQR: 62–71) ^b^	Germany, Canada	Retrospective cohort	Recurrent disease	111
12 [31]	Mayr, 2024	70 (IQR 65–73) ^b^	Germany	Retrospective cohort	Recurrent disease	50
13 [32]	Harke, 2024	72 (range: 61–80) ^b^	Germany	Retrospective cohort	Primary intermediate -/high-risk	12
14 [33]	Quarta, 2024	68 (IQR: 62–70) ^b^	Italy	Prospective cohort	Primary high-risk	30
15 [34]	Collamati, 2024	63 (IQR: 53–68) ^b^	Netherlands/Italy	Prospective cohort	Primary high-risk	7
16 [35]	Schilham, 2024	69 (range: 57–79) ^b^	Netherlands	Prospective cohort	Primary intermediate -/high-risk	20
17 [36]	Ambrosini, 2024	OPEN: 63 (IQR: 60–69) ^b^; RA: 64 (IQR: 60–67) ^b^	Germany	Retrospective cohort	Recurrent disease	85
18 [37]	Winkens, 2023	55, 66, 82, 73, 73, 62 ^c^	Germany	Retrospective cohort	Recurrent disease	6
19 [18]	Lunger, 2023	66 (IQR: 64–69) ^b^	Germany	Retrospective cohort	Primary intermediate -/high-risk	35
20 [38]	Knipper, 2021	67 (IQR: 63–74) ^b^	Germany	Retrospective cohort	Recurrent disease	40
21 [39]	Darr, 2020	72 (median) ^b^	Germany	Prospective cohort	Primary high-risk	10
22 [40]	Heuvel, 2020	67, 71, 58, 73, 63^c^	Netherlands	Prospective cohort	Primary high-risk	5
23 [41]	Heuvel, 2022	65.6 (IQR: 60.8–70.5) ^b^	Netherlands	Prospective cohort	Primary high-risk	15
24 [42]	Darr, 2021	66 (IQR: 59–69) ^b^	Germany	Prospective cohort	Primary intermediate -/high-risk	10
25 [43]	Muraglia, 2023	NR	Italy	Prospective cohort	Primary high-risk	2
26 [15]	Darr, 2023	65.6 (IQR: 60.8–70.5) ^b^	Germany	Prospective cohort	Primary high-risk	10
27 [44]	Moraitis, 2025	68 (range: 60–80) ^b^	Germany	Prospective cohort	Primary high-risk	7
28 [45]	Mazzucato, 2024	71 (IQR: 66–72)^b^	Germany	Retrospective cohort	Recurrent disease	13

ᵃ Reported as mean ± SD in the original article

ᵇ Reported as median (IQR) or median (range) in the original article

ᶜ individual values

#### Surgical procedures and radioguided parameters

The surgical and radioguided procedures exhibited notable heterogeneity across the included studies. Most patients underwent either open or robot-assisted radical prostatectomy with pelvic lymph node dissection, though salvage procedures were also described.Radioguidance was primarily achieved using γ-probes, with occasional use of β-probes and fluorescence/Cerenkov imaging systems. A range of PSMA-targeting tracers were applied, including [^68^ Ga]Ga-PSMA-11, [^99m^Tc]Tc-PSMA-I&S, [^111^In]In-PSMA-I&T, and [^18^F]PSMA-1007, with intraoperative doses ranging from 3.2 to 735 MBq. Major complications (Clavien-Dindo grade ≥ III) occurred in up to 12.5% of cases, most commonly lymphorrhea and surgical site injury. Postoperative adjuvant therapies were frequently reported. Details of specific tracers, detection methods, complications, and treatments by study are provided in Supplementary Table S5.

#### Pathological outcomes and prior therapies

The baseline pathological characteristics and prior treatment histories of the patient cohorts are summarized in Supplementary Table S6. There was substantial heterogeneity in disease aggressiveness, as reflected by a wide spectrum of ISUP grades and pathological T-stages. The time from previous local treatment (e.g., radical prostatectomy or radiotherapy) to radioguided surgery also varied extensively, ranging from months to years. Approximately one-third of the studies enrolled only treatment-naïve patients, while the remainder included populations with varying histories of prior therapy.

### Diagnostic performance

As shown in Table 2, the diagnostic performance of PSMA-targeted imaging varied across analysis levels (patient, lesion, region, and lymph node) and imaging timepoints (preoperative imaging, intraoperative in vivo RGS, and ex vivo RGS).

**Table 2:** Diagnostic performance of imaging modalities at different analysis levels and time points

Analysis Levels	Imaging Modalities	Sensitivity (95% CI)	Specificity (95% CI)
Patient level	Preoperative Imaging	97% (85, 99)	38% (07, 84)
	In vivo RGS	94% (81, 98)	92% (83, 96)
	Ex vivo RGS	87% (79, 93)	98% (93, 99)
Lesion level	Preoperative Imaging	89% (74, 96)	44% (07, 89)
	In vivo RGS	90% (78, 96)	97% (88, 99)
	Ex vivo RGS	87% (79, 93)	100% (98, 100)
Region level	Preoperative Imaging	68% (59, 76)	95% (10, 100)
	In vivo RGS	71% (50, 86)	90% (71, 97)
	Ex vivo RGS	68% (59, 76)	95% (10, 100)
Lymph nodes level	In vivo RGS	97% (89, 99)	99% (93, 100)
	Ex vivo RGS	93% (82, 97)	100% (98, 100)

In general, ex vivo RGS imaging consistently demonstrated higher specificity (ranging from 95 to 100%) compared to preoperative imaging or in vivo RGS modalities. At the patient level, sensitivity decreased slightly across the surgical timeline (97% pre-op RGS vs. 94% in vivo RGS vs. 87% ex vivo RGS), while specificity improved markedly from 38% preoperative imaging to 98% ex vivo RGS. At the lesion level, both in vivo RGS and ex vivo RGS imaging achieved high sensitivity (90% and 87%, respectively) and excellent specificity (97–100%). The region-level analysis revealed lower sensitivities (68–71%) but retained high specificity (90–95%). It is noteworthy that the lymph node-level analysis exhibited optimal diagnostic accuracy, with sensitivity levels of 97% (in vivo RGS) and 93% (ex vivo RGS), and specificity levels of 100% in both settings.

#### Patient-level analysis

**Fig. 3:**
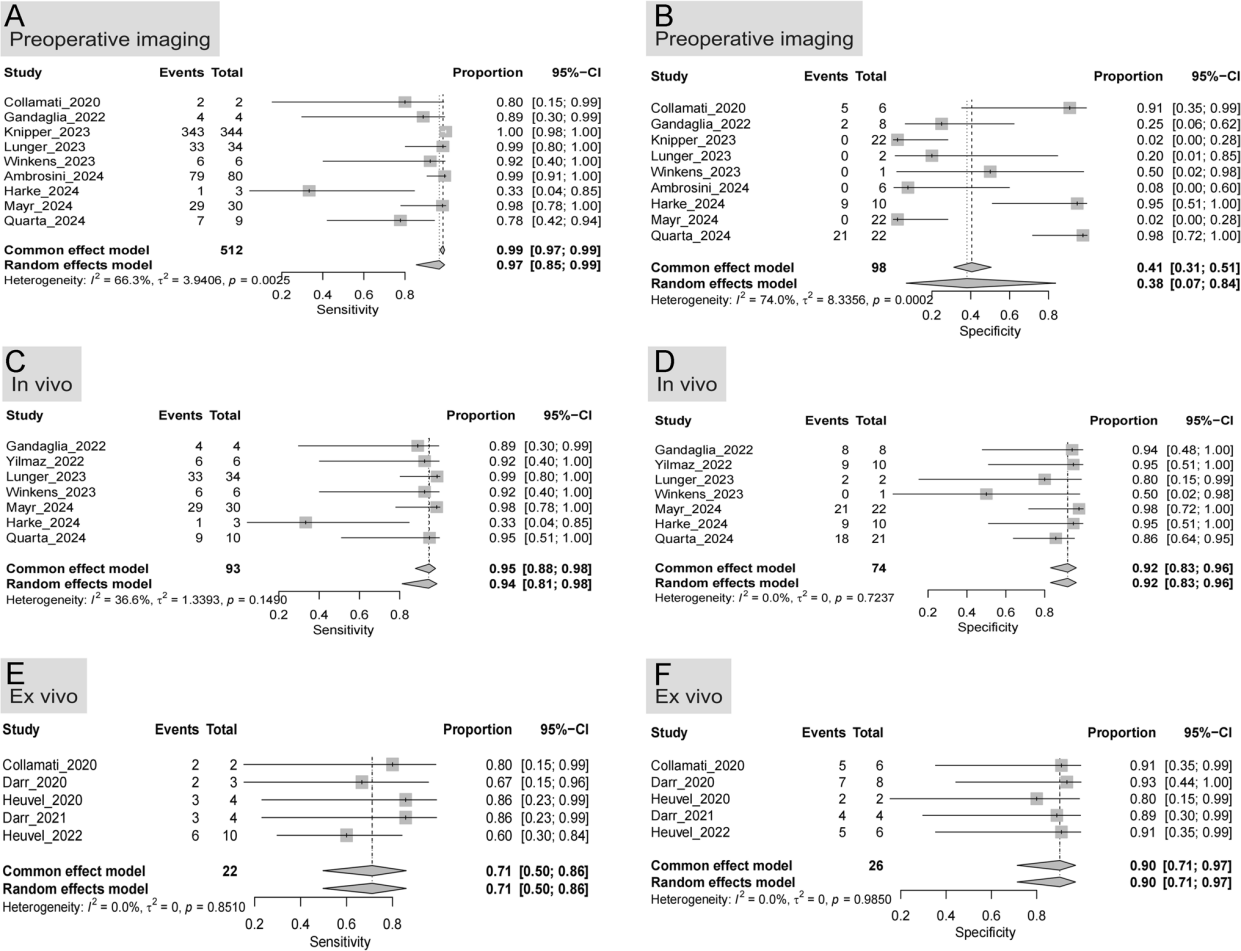
Forest plots at patient level—diagnostic performance. (**A**–**B**) Preoperative imaging: pooled sensitivity (A) and specificity (B); (**C**–**D**) In vivo PSMA-RGS: pooled sensitivity (C) and specificity (D); (**E**–**F**) Ex vivo PSMA-RGS: pooled sensitivity (E) and specificity (F)

At the patient level (Fig. 3), preoperative imaging yielded pooled sensitivities and specificities of 97% (95% CI: 85–99) and 38% (95% CI: 07–84) respectively, with moderate heterogeneity (I^2^ = 66.3%, I^2^ = 74.0%). In vivo RGS demonstrated high sensitivity (95%, 88–98) and specificity (92%, 83–96), with low heterogeneity. Ex vivo RGS also achieved excellent sensitivity 87% (79–93) and specificity 98% (93–99) with I^2^ = 0%. The corresponding Fagan nomograms and HSROC curves at the patient level are presented in Supplementary Figures S2 and S3.

#### Lesion-level analysis

Forest plots for lesion-level analyses are provided in Supplementary Figure S4, preoperative imaging showed a pooled sensitivity of 89% (74–96%), but a relatively low specificity of 44% (0.07–0.89%). In vivo RGS achieved a sensitivity of 90% (78–96) and a specificity of 97% (88–99), with negligible heterogeneity. Ex vivo RGS had similar performance: sensitivity 87% (79–93) and specificity 100% (98–100), indicating robust diagnostic accuracy.To further evaluate the clinical interpretability of diagnostic performance, the scatter plot of log positive likelihood ratio (PLR) versus log negative likelihood ratio (NLR) at the lesion level is provided in Supplementary Figure S5.

#### Region-level analysis

Forest plots for region-level analyses are provided in Supplementary Figure S6. Preoperative imaging showed sensitivity 68% (59–76) and specificity 95% (10–100) with significant heterogeneity (I^2^ > 68%). In vivo RGS improved both sensitivity 71% (50–86) and specificity 90% (71–97). Ex vivo RGS maintained sensitivity at 68% (59–76) and specificity at 95% (10–100), with minimal heterogeneity.

#### Lymph node-level analysis

Forest plots for lymph node–level analyses are provided in Supplementary Figure S7. A lymph node-based analysis revealed high accuracy for both in vivo and ex vivo RGS modalities. In vivo RGS achieved a sensitivity of 97% (89–99%) and a specificity of 96% (93–99%), with acceptable heterogeneity (I^2^ = 56.8–77%). Ex vivo RGS yielded sensitivity 90% (85–93) and specificity 100% (98–100), demonstrating optimal performance with low heterogeneity.

To summarize global discriminatory performance across modalities at the patient level, pooled positive likelihood ratios and diagnostic odds ratios are presented in Supplementary Figures S8–S9.

### Subgroup and meta-regression analysis

Supplementary Table S7 summarizes the subgroup analyses stratified by modality, anatomical level, study design, and probe type. PSMA-RGS, particularly the in vivo approach, showed significantly higher pooled log diagnostic odds ratio (logDOR^1^) compared to preoperative imaging (5.77 vs. 2.99; *p* < 0.0001).Lymph node–based analysis yielded the highest diagnostic performance (log DOR = 7.08; *p* = 0.0011 vs. lesion-based).

Studies using γ-probes with germanium detectors were analyzed as a separate group due to their distinct technical properties and post-operative measurement setting [22, 34]. Among intraoperative probes, the γ-probe combined with germanium detection and VisionSense NIR system demonstrated significantly improved diagnostic performance (*p* = 0.0111 and *p* = 0.0231, respectively). This performance advantage may relate to the superior energy resolution of high-purity germanium detectors, which enables clearer differentiation of PSMA-specific signals from background noise, especially in low-count environments or anatomically complex regions.

Supplementary Table S8 shows the results of meta-regression modeling. Independent predictors of improved diagnostic performance included RGS in vivo (estimate = 2.37, 95% CI: 1.21–3.53, *p* < 0.0001), RGSex vivo (estimate = 1.59, *p* = 0.0313), lymph node–based analysis (1.69, *p* = 0.0235), and use of γ-probe plus germanium detector (2.61, *p* = 0.0126). Use of fluorescence was not statistically significant but showed a trend toward improved performance.However, this covariate was represented by a single feasibility study [29], and results should therefore be interpreted as exploratory only.

Robustness checks using leave-one-out analyses are shown in Supplementary Figures S10–S12, indicating stable pooled estimates.

### Publication bias

Publication bias was evaluated using funnel plots at the patient level. Visual inspection suggested potential asymmetry for sensitivity, which indicated possible publication bias. After applying the trim-and-fill method, the asymmetry was attenuated, suggesting that the results were robust (Supplementary Figures S13–S14). In contrast, funnel plots for specificity showed no evidence of asymmetry, and no correction was required (Supplementary Figure S15).

## Discussion

This systematic review and meta-analysis demonstrate that PSMA-RGS achieves high diagnostic accuracy for lesion identification and consistently outperforms preoperative imaging modalities such as PSMA PET/CT or SPECT. Specificity reached 92–100% compared with 38–44% for imaging, while sensitivity remained excellent (87–94%), highlighting the potential value of real-time intraoperative guidance in improving tumor localization and resection completeness.

Across tracers, ⁹⁹ᵐTc-labelled PSMA-I&S was the most frequently used and showed reliable intraoperative γ-probe guidance; ^111^In-labelled ligands were less common with comparable performance, whereas ⁶⁸Ga-labelled agents were mainly confined to preoperative imaging or exploratory intraoperative applications [10, 12]. By restricting inclusion to studies using PSMA-targeted tracers, this meta-analysis minimized potential confounding and enabled a focused evaluation of diagnostic performance.

PSMA-based image guidance can be divided into preoperative imaging, intraoperative in vivo RGS, and ex vivo RGS. Preoperative imaging refers to PSMA PET or SPECT performed before surgery to localize suspected lesions and support surgical planning, without providing real-time intraoperative feedback. Intraoperative in vivo RGS involves real-time detection of PSMA-targeted radiotracer uptake within the patient during surgery using a handheld gamma probe, enabling lesion identification beyond visual or tactile guidance, whereas ex vivo RGS is performed on resected specimens to quantify probe signal intensity and directly correlate radiotracer uptake with histopathological findings [10, 12, 16].

Owing to the controlled measurement conditions and the absence of background activity, ex vivo RGS is most commonly regarded as the reference standard for assessing detection accuracy in PSMA-RGS studies [12, 13], and consequently yields higher detection or confirmation rates than intraoperative in vivo measurements. In contrast, in vivo RGS, while clinically valuable for real-time surgical decision-making, is inherently more susceptible to background activity and anatomical constraints and is therefore commonly validated against ex vivo findings. These differences underscore the complementary roles of the two approaches, with ex vivo assessment serving primarily as a validation benchmark rather than a decision-directing tool.

Compared to prior studies solely evaluating PSMA PET imaging [46], our meta-analysis shows that intraoperative PSMA-RGS—especially when confirmed with ex vivo measurements—may improve sensitivity for small or sub-centimeter lesions, including those below the detection threshold of conventional imaging modalities [14, 19]. In addition, one study confirmed the feasibility of a DROP-IN γ-probe with [^99m^Tc]Tc-PSMA-I&S during robot-assisted prostatectomy [14]. Germanium detectors were mainly used post-resection as confirmation tools [22], and were analyzed separately to reflect differences in timing and workflow.

Significant heterogeneity was observed in the preoperative imaging subgroups (I^2^ = 74.0% at the patient level), mainly reflecting variability in radiotracer types, imaging protocols, and patient risk stratification. Differences in PSMA ligands, imaging resolution, and interpretation criteria likely contributed to divergent sensitivity estimates. In contrast, intraoperative RGS showed lower heterogeneity (I^2^ < 35%), probably owing to more standardized surgical workflows and γ-probe procedures across centers (see Supplementary Table S6).

The clinical role of PSMA-RGS appears to differ between primary surgery in high-risk prostate cancer and salvage surgery performed in the setting of biochemical recurrence. In patients undergoing salvage lymph node dissection for biochemical recurrence, PSMA-RGS has been shown to facilitate targeted identification of limited metastatic lesions, particularly in cases with uni- or bifocal recurrence detected on preoperative PSMA imaging, where precise intraoperative localization may directly translate into complete resection of all known disease.

By contrast, in the primary high-risk setting, PSMA-RGS is most often applied in conjunction with extended pelvic lymph node dissection, where its incremental benefit over systematic template-based dissection remains less well defined and is supported by more limited clinical evidence [6, 17].

The added value of PSMA-RGS also depends on the anatomical localization of lymph node metastases in relation to established dissection templates. In standard pelvic nodal regions, such as the obturator and external iliac areas, systematic dissection alone already achieves high detection rates, potentially limiting the incremental contribution of radioguided guidance. Conversely, PSMA-RGS may be particularly advantageous for lesions located outside or at the margins of conventional templates, including presacral, common iliac, or other atypical nodal stations, where visual identification is more challenging and lesion-directed intraoperative localization can facilitate selective resection [6, 25]. This pattern is frequently observed in the salvage setting, in which PSMA imaging often reveals non–template-based distribution and limited disease burden [6, 10, 17].

Although definitive patient selection criteria cannot yet be established, consistent patterns across included studies suggest that PSMA-RGS may offer the greatest clinical benefit in patients with biochemical recurrence, low PSA levels, and limited nodal disease burden. Gleason score and multiparametric MRI findings should be regarded as complementary rather than exclusive determinants, as the effectiveness of PSMA-RGS is primarily driven by PSMA expression and lesion detectability rather than histologic grade alone [11, 16].

The potential effectiveness of PSMA-RGS may further vary between open and minimally invasive surgical approaches. In open surgery, direct tactile feedback and wide exposure already facilitate lymph node identification, potentially limiting the incremental benefit of radioguided assistance. In contrast, minimally invasive and robot-assisted procedures lack tactile feedback and operate within a constrained visual field, where PSMA-RGS—particularly with drop-in or robotic-compatible gamma probes—may provide added value by enabling precise localization of PSMA-avid lesions beyond visual guidance alone. Nevertheless, direct comparative data remain limited, precluding definitive conclusions regarding superiority of one surgical approach over another [7, 14].

Several limitations warrant acknowledgment. First, the majority of included studies were retrospective, single-center investigations with small sample sizes and predominantly non-randomized designs. Second, technical parameters varied across studies, including radiotracer injection doses, probe types, and imaging protocols. Third, patient outcome metrics were inconsistent (e.g., survival rates and disease-free survival), and limited follow-up periods with insufficient long-term survival data constrained interpretation of PSMA-RGS long-term therapeutic efficacy.Despite these limitations, subgroup and meta-regression analyses (Supplementary Table S7 and S8) confirmed that imaging modality and anatomical level strongly influence diagnostic performance. In particular, in vivo RGS and lymph node–based analyses achieved the highest log DOR, and the γ-probe plus germanium detector was an independent predictor of improved accuracy (*p* = 0.0126). These findings support the robustness of intraoperative radioguided approaches while underscoring the importance of technical standardization.

Future investigations should prioritize prospective, multicenter controlled trials to evaluate the long-term clinical benefits of PSMA-RGS, including its genuine impact on lymph node dissection adequacy, biochemical recurrence rates, and survival endpoints. Study designs should incorporate comparative surgical approaches (conventional ePLND vs. PSMA-RGS), standardized radiotracer protocols, unified probe parameters, and intraoperative feedback mechanisms.

In parallel, integration of PSMA-radioguided surgery with multimodal intraoperative imaging techniques, such as fluorescence or Cerenkov imaging, may further enhance lesion detection efficiency. While fluorescence-guided surgery offers high visual resolution, its clinical utility is constrained by limited tissue penetration, whereas radioguided techniques enable deeper signal detection and real-time intraoperative feedback [39, 47].

However, broader implementation of radioguided approaches remains dependent on radiochemistry infrastructure, isotope logistics, and regulatory considerations, which currently restrict their use to specialized centers. Hybrid dual-labeled tracers, such as PSMA-I&F, represent a promising strategy to combine the complementary strengths of radioactive and optical imaging and may further improve surgical accuracy, particularly in minimally invasive and robot-assisted procedures. PSMA-targeted RGS is a promising intraoperative adjunct for precision surgery, with growing evidence supporting its role in lesion localization and surgical guidance, while highlighting the need for further high-quality evidence to define its place in routine clinical practice.

## Conclusion

PSMA-RGS has shown promising diagnostic accuracy, particularly for lymph node metastases, and may enhance intraoperative lesion localization through real-time γ-probe guidance. This approach may help identify previously unrecognized nodal involvement intraoperatively, potentially altering the perceived extent of disease and guiding more complete resection. Its integration into surgical workflows may improve resection completeness and reduce recurrence in high-risk prostate cancer. However, the current evidence is based on relatively small and heterogeneous studies, and long-term oncologic outcomes remain uncertain. Despite these limitations, PSMA-RGS represents a promising adjunct to precision surgery. Future research should prioritize prospective multicenter trials and standardized reporting to define its role in routine surgical practice.

## Supplementary Information

Below is the link to the electronic supplementary material.

Supplementary file1 (DOCX 432 KB)

Supplementary file2 (DOCX 91 KB)

Supplementary file3 (DOCX 210 KB)

Supplementary file4 (DOCX 266 KB)

Supplementary file5 (DOCX 432 KB)

Supplementary file6 (DOCX 432 KB)

Supplementary file7 (DOCX 211 KB)

Supplementary file8 (DOCX 377 KB)

Supplementary file9 (DOCX 309 KB)

Supplementary file10 (DOCX 386 KB)

Supplementary file11 (DOCX 333 KB)

Supplementary file12 (DOCX 346 KB)

Supplementary file13 (DOCX 106 KB)

Supplementary file14 (DOCX 73 KB)

Supplementary file15 (DOCX 82 KB)

Supplementary file16 (DOCX 16 KB)

Supplementary file17 (DOCX 13 KB)

Supplementary file18 (DOCX 13 KB)

Supplementary file19 (DOCX 68.5 KB)

Supplementary file20 (DOCX 74.4 KB)

Supplementary file21 (DOCX 75.0 KB)

Supplementary file22 (DOCX 18 KB)

Supplementary file23 (DOCX 14 KB)

## Data Availability

All data analysed in this study were extracted from previously published articles included in the systematic review. No new datasets were generated. Additional information is available from the corresponding author upon reasonable request.

## Footnotes

1 Note: The diagnostic odds ratio (DOR), particularly expressed on the logarithmic scale (log DOR), is a global measure of diagnostic test performance that combines both sensitivity and specificity into a single metric. Higher log DOR values indicate stronger diagnostic discrimination.
