# Supplementary material for: Diagnostic accuracy of PSMA-targeted radioguided surgery in prostate cancer at multiple anatomical levels: a systematic review and meta-analysis
Source: Eur J Nucl Med Mol Imaging. 2026 Mar 27;53(8):4850–61. doi: 10.1007/s00259-026-07773-x (PMC13249658; doi:10.1007/s00259-026-07773-x)

**Article Title:**

Diagnostic Accuracy of PSMA-Targeted Radioguided Surgery in Prostate Cancer at Multiple Anatomical Levels: A Systematic Review and Meta-analysis

**Journal:**

European Journal of Nuclear Medicine and Molecular Imaging (EJNMMI)

**Authors:**

Fang Wen, Laura Schäfer, Xinlin Zheng, Hao Huang, Walter Noordzij, Matthias Saar, Felix M. Mottaghy, Susanne Lütje

**Corresponding Author:**

Univ.-Prof. Dr. Dr. med. Susanne Lütje

Department of Nuclear Medicine

University Hospital RWTH Aachen

Pauwelsstraße 30

52074 Aachen

Germany

Email: sluetje@ukaachen.de

**File Type:**

Supplementary Material – Supplementary Figure S8


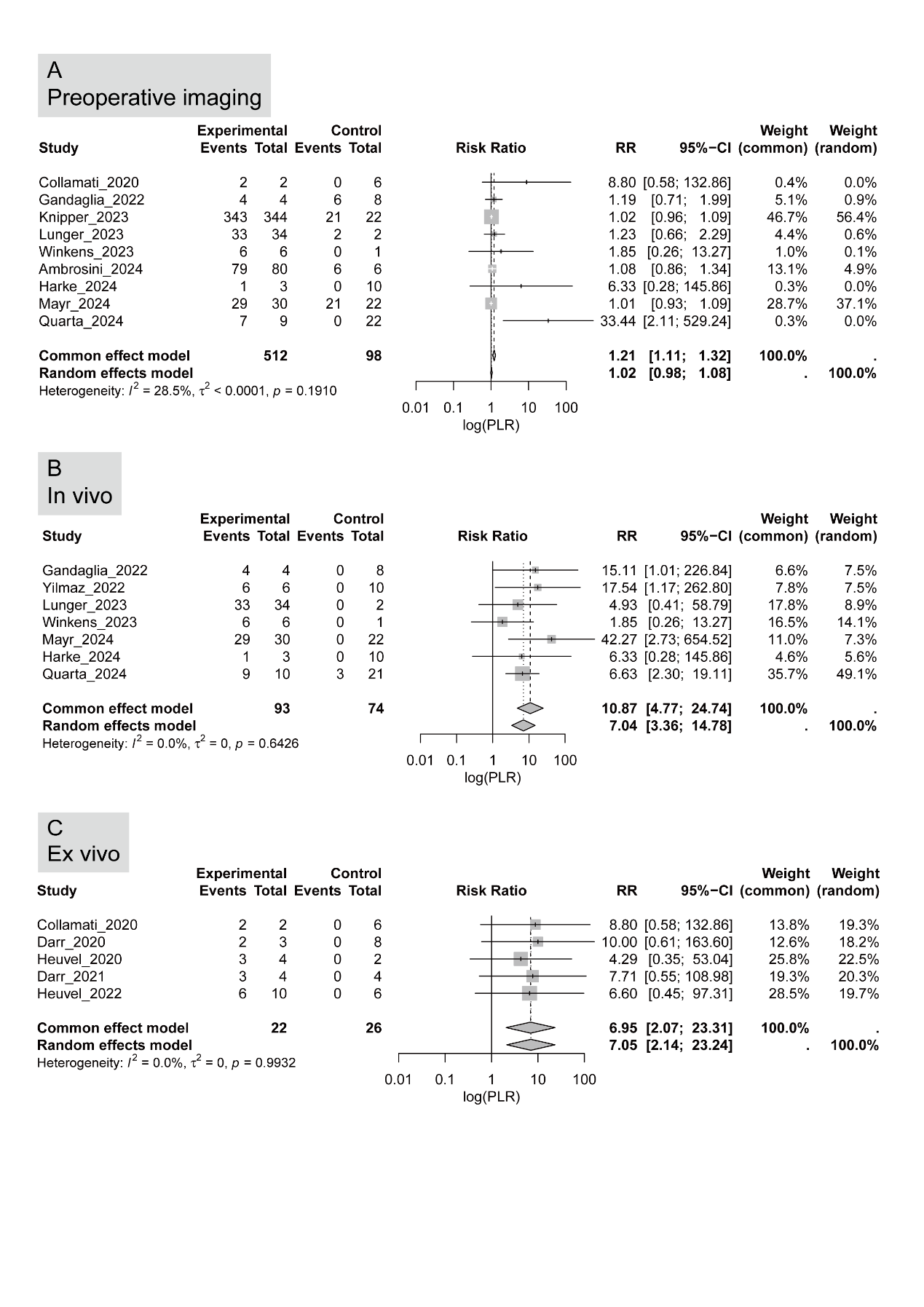

Supplement: Supplementary file 8 — Supplementary file8 (DOCX 377 KB) [file 259_2026_7773_MOESM8_ESM.docx]
