# Supplementary material for: Diagnostic accuracy of PSMA-targeted radioguided surgery in prostate cancer at multiple anatomical levels: a systematic review and meta-analysis
Source: Eur J Nucl Med Mol Imaging. 2026 Mar 27;53(8):4850–61. doi: 10.1007/s00259-026-07773-x (PMC13249658; doi:10.1007/s00259-026-07773-x)
Supplement: Supplementary file 16 — Supplementary file16 (DOCX 16 KB) [file 259_2026_7773_MOESM16_ESM.docx]

**Article Title:**

Diagnostic Accuracy of PSMA-Targeted Radioguided Surgery in Prostate Cancer at Multiple Anatomical Levels: A Systematic Review and Meta-analysis

**Journal:**

European Journal of Nuclear Medicine and Molecular Imaging (EJNMMI)

**Authors:**

Fang Wen, Laura Schäfer, Xinlin Zheng, Hao Huang, Walter Noordzij, Matthias Saar, Felix M. Mottaghy, Susanne Lütje

**Corresponding Author:**

Univ.-Prof. Dr. Dr. med. Susanne Lütje

Department of Nuclear Medicine

University Hospital RWTH Aachen

Pauwelsstraße 30

52074 Aachen

Germany

Email: sluetje@ukaachen.de

**File Type:**

Supplementary Material – Supplementary Table S1

**Supplementary Table S1.** Search strategy for PubMed database

| **Search Concept** | **Search strategy** | |
| --- | --- | --- |
| **Prostate cancer** | #1 | (Prostatic Neoplasms[MeSH]) OR ((prostate[Title/Abstract] OR prostatic [Title/Abstract])  AND (neoplasm* [Title/Abstract] OR cancer* [Title/Abstract] OR tumor*[Title/Abstract]  OR tumour*[Title/Abstract] OR carcinoma*[Title/Abstract] OR malignan*[Title/Abstract]  OR adenocarci-noma*[Title/Abstract])) |
| **Surgery** | #2 | (("surgical procedures, operative"[MeSH] OR "prostatectomy"[MeSH] OR "lymph node excision"[MeSH])  OR (surger*[Title/Abstract] OR surgical*[Title/Abstract] OR prostatectom*[Title/Abstract]  OR salvage[Title/Abstract] OR ("Robotic Surgical Procedures"[MeSH] OR robot*[Title/Abstract])  OR ((("lymph node*"[Title/Abstract]) OR (("sentinel node*"[Title/Abstract])) AND (dissection*[Title/Abstract] OR biops*[Title/Abstract] OR excision*[Title/Abstract])) OR lymphadenectomy[Title/Abstract]))  OR "intraoperative" [Title/Abstract] |
| **Radioguided or Imaging-Guided** | #3 | ("Positron Emission Tomography"[MeSH] OR "Tomography, Emission Computed"[MeSH]  OR "Positron Emission Tomography Computed Tomography"[MeSH]) OR ("PET/CT"[Title/Abstract]  OR "PET CT"[Title/Abstract]OR "PET Computed Tomography"[Title/Abstract] OR"Fluorescence"[Title/Abstract] OR "CLI"[Title/Abstract] ) OR (Radiopharmaceuticals[MesH])  OR (Fluorescence[MeSH]) OR "Radioguided"[Title/Abstract]OR "Radio guided"[Title/Abstract]  OR "Radioguidance"[Title/Abstract] OR "Radio guidance"[Title/Abstract] OR "Imaging*"[Title/Abstract]  OR "gamma"[Title/Abstract] AND (("prostate specific membrane antigen"[Title/Abstract])  OR "PSMA"[Title/Abstract]) |
| **Combined strategy** | #4 | #1 AND #2 AND #3 |
