# Supplementary material for: Diagnostic accuracy of PSMA-targeted radioguided surgery in prostate cancer at multiple anatomical levels: a systematic review and meta-analysis
Source: Eur J Nucl Med Mol Imaging. 2026 Mar 27;53(8):4850–61. doi: 10.1007/s00259-026-07773-x (PMC13249658; doi:10.1007/s00259-026-07773-x)
Supplement: Supplementary file 17 — Supplementary file17 (DOCX 13 KB) [file 259_2026_7773_MOESM17_ESM.docx]

**Article Title:**

Diagnostic Accuracy of PSMA-Targeted Radioguided Surgery in Prostate Cancer at Multiple Anatomical Levels: A Systematic Review and Meta-analysis

**Journal:**

European Journal of Nuclear Medicine and Molecular Imaging (EJNMMI)

**Authors:**

Fang Wen, Laura Schäfer, Xinlin Zheng, Hao Huang, Walter Noordzij, Matthias Saar, Felix M. Mottaghy, Susanne Lütje

**Corresponding Author:**

Univ.-Prof. Dr. Dr. med. Susanne Lütje

Department of Nuclear Medicine

University Hospital RWTH Aachen

Pauwelsstraße 30

52074 Aachen

Germany

Email: sluetje@ukaachen.de

**File Type:**

Supplementary Material – Supplementary Table S2

**Supplementary Table S2.** Search strategy for Embase database

| **Search Concept** | **Search strategy** | |
| --- | --- | --- |
| **Prostate Cancer** | #1 | ('prostatic neoplasm'/exp OR ((prostate:ti,ab,kw OR prostatic:ti,ab,kw) AND (neoplasm*:ti,ab,kw  OR cancer*:ti,ab,kw OR tumor*:ti,ab,kw OR tumour*:ti,ab,kw OR carcinoma*:ti,ab,kw  OR malignan*:ti,ab,kw OR adenocarcinoma*:ti,ab,kw))) |
| **Surgery** | #2 | ('surgical procedure'/exp OR 'prostatectomy'/exp OR 'lymph node excision'/exp) OR (surger*:ti,ab,kw OR surgical*:ti,ab,kw OR prostatectom*:ti,ab,kw OR salvage:ti,ab,kw  OR ('robot assisted surgery'/exp OR robot*:ti,ab,kw) OR (('lymph node*':ti,ab,kw OR 'sentinel node*':ti,ab,kw) AND (dissection*:ti,ab,kw OR biops*:ti,ab,kw OR excision*:ti,ab,kw)  OR lymphadenectomy:ti,ab,kw)) OR 'intraoperative':ti,ab,kw |
| **Radioguided or Imaging-Guided** | #3 | (('positron emission tomography'/exp OR 'emission computed tomography'/exp OR 'positron emission tomography computed tomography'/exp) OR ('PET/CT':ti,ab,kw OR 'PET CT':ti,ab,kw  OR 'PET computed tomography':ti,ab,kw OR 'fluorescence':ti,ab,kw OR 'CLI':ti,ab,kw)  OR 'radiopharmaceutical'/exp OR 'fluorescence'/exp OR ('radioguided':ti,ab,kw  OR 'radio guided':ti,ab,kw OR 'radioguidance':ti,ab,kw OR 'radio guidance':ti,ab,kw OR imaging*:ti,ab,kw OR gamma:ti,ab,kw) AND ('prostate specific membrane antigen'/exp  OR 'PSMA':ti,ab,kw)) |
| **Combined strategy** | #4 | #1 AND #2 AND #3 |
