# Supplementary material for: Diagnostic accuracy of PSMA-targeted radioguided surgery in prostate cancer at multiple anatomical levels: a systematic review and meta-analysis
Source: Eur J Nucl Med Mol Imaging. 2026 Mar 27;53(8):4850–61. doi: 10.1007/s00259-026-07773-x (PMC13249658; doi:10.1007/s00259-026-07773-x)
Supplement: Supplementary file 18 — Supplementary file18 (DOCX 13 KB) [file 259_2026_7773_MOESM18_ESM.docx]

**Article Title:**

Diagnostic Accuracy of PSMA-Targeted Radioguided Surgery in Prostate Cancer at Multiple Anatomical Levels: A Systematic Review and Meta-analysis

**Journal:**

European Journal of Nuclear Medicine and Molecular Imaging (EJNMMI)

**Authors:**

Fang Wen, Laura Schäfer, Xinlin Zheng, Hao Huang, Walter Noordzij, Matthias Saar, Felix M. Mottaghy, Susanne Lütje

**Corresponding Author:**

Univ.-Prof. Dr. Dr. med. Susanne Lütje

Department of Nuclear Medicine

University Hospital RWTH Aachen

Pauwelsstraße 30

52074 Aachen

Germany

Email: sluetje@ukaachen.de

**File Type:**

Supplementary Material – Supplementary Table S3

**Supplementary Table S3.** Search strategy f**or Web of Science database**

| **Search Concept** | **Search strategy** | |
| --- | --- | --- |
| **Prostate Cancer** | #1 | TS=("prostate cancer" OR "prostatic neoplasm" OR "prostatic carcinoma" OR "prostatic adenocarcinoma" OR "malignant prostate tumor") |
| **Surgery** | #2 | TS=("prostatectomy" OR "surgical procedure" OR "lymph node excision" OR "robot-assisted surgery" OR "sentinel node biopsy" OR "lymphadenectomy" OR "salvage surgery" OR "intraoperative") |
| **Radioguided or Imaging-Guided** | #3 | TS=("positron emission tomography" OR "PET/CT" OR "PET-MRI" OR "fluorescence imaging" OR "radioguided surgery" OR "radioguidance" OR "radio guided surgery" OR "gamma imaging" OR "radiopharmaceutical" OR "PSMA-targeted imaging" OR "prostate specific membrane antigen") |
| **Combined strategy** | #4 | #1 AND #2 AND #3 |
