# Supplementary material for: Diagnostic accuracy of PSMA-targeted radioguided surgery in prostate cancer at multiple anatomical levels: a systematic review and meta-analysis
Source: Eur J Nucl Med Mol Imaging. 2026 Mar 27;53(8):4850–61. doi: 10.1007/s00259-026-07773-x (PMC13249658; doi:10.1007/s00259-026-07773-x)
Supplement: Supplementary file 19 — Supplementary file19 (DOCX 68.5 KB) [file 259_2026_7773_MOESM19_ESM.docx]

**Article Title:**

Diagnostic Accuracy of PSMA-Targeted Radioguided Surgery in Prostate Cancer at Multiple Anatomical Levels: A Systematic Review and Meta-analysis

**Journal:**

European Journal of Nuclear Medicine and Molecular Imaging (EJNMMI)

**Authors:**

Fang Wen, Laura Schäfer, Xinlin Zheng, Hao Huang, Walter Noordzij, Matthias Saar, Felix M. Mottaghy, Susanne Lütje

**Corresponding Author:**

Univ.-Prof. Dr. Dr. med. Susanne Lütje

Department of Nuclear Medicine

University Hospital RWTH Aachen

Pauwelsstraße 30

52074 Aachen

Germany

Email: sluetje@ukaachen.de

**File Type:**

Supplementary Material – Supplementary Table S4

**Supplementary Table S4.** Characteristics of Included Studies(Study and Patient Characteristics)

| **Ref** | **Author, year** | **Age (years)** | **Country** | **Center Type** | **Study Design** | **Blinding** | **Patient Type** | **Sample Size (n)** |
| --- | --- | --- | --- | --- | --- | --- | --- | --- |
| 1 [21] | Collamati, 2020 | 71,57,73,66,63,55,48^c^ | Netherlands / Italy | Single-center | Prospective cohort | Open-label | Primary high-risk | 7 |
| 2 [22] | Jilg, 2020 | 67.5±6.6 ^a^ | Germany | Single-center | Retrospective cohort | Open-label | Recurrent Disease or primary cN+ | 23 |
| 3 [23] | Mix,2021 | 61.0 ± 8.0 ^a^ | Germany | Single-center | Prospective cohort | Open-label | Recurrent Disease or primary cN+ | 6 |
| 4 [14] | de Barros, 2022 | Median 68 (IQR 66–72)^b^ | Netherlands | Single-center | Prospective cohort | Open-label | Recurrent disease | 20 |
| 5 [24] | Gondoputro, 2022 | Median 68 (IQR 57–69) ^b^ | Australia | Single-center | Prospective cohort | Open-label | Primary high-risk | 12 |
| 6 [25] | Knipper, 2023 | 67 (IQR: 62–71) ^b^ | Germany | Multicenter | Retrospective cohort | Open-label | Recurrent disease | 364 |
| 7 [26] | Yılmaz, 2022 | 63.3 ± 6.2 ^a^ | Turkey | Multicenter | Prospective cohort | Open-label | Primary intermediate -/ high-risk | 15 |
| 8 [27] | Gandaglia,2022 | 70 (IQR: 66–71) ^b^ | Italy | Single-center | Prospective cohort | Open-label | Primary intermediate -/ high-risk | 12 |
| 9 [28] | Koehler, 2023 | Median 62 (IQR 61–67) ^b^ | Germany | Single-center | Retrospective cohort | Open-label | Recurrent disease | 9 |
| 10 [29] | Stibbe, 2023 | 69 (IQR: 64–70) ^b^ | Netherlands | Single-center | Prospective cohort | Open-label | Primary intermediate -/ high-risk | 18 |
| 11 [30] | Falkenbach, 2025 | 67 (IQR: 62–71) ^b^ | Germany, Canada | Multicenter | Retrospective cohort | Open-label | Recurrent disease | 111 |
| 12 [31] | Mayr, 2024 | 70 (IQR 65–73) ^b^ | Germany | Single-center | Retrospective cohort | Open-label | Recurrent disease | 50 |
| 13 [32] | Harke, 2024 | 72 (range: 61–80) ^b^ | Germany | Single-center | Retrospective cohort | Open-label | Primary intermediate -/ high-risk | 12 |
| 14 [33] | Quarta, 2024 | 68 (IQR: 62–70) ^b^ | Italy | Single-center | Prospective cohort | Open-label | Primary high-risk | 30 |
| 15 [34] | Collamati, 2024 | 63 (IQR: 53–68) ^b^ | Netherlands / Italy | Single-center | Prospective cohort | Open-label | Primary high-risk | 7 |
| 16 [35] | Schilham, 2024 | 69 (range: 57–79) ^b^ | Netherlands | Multicenter | Prospective cohort | Open-label | Primary intermediate -/ high-risk | 20 |
| 17 [36] | Ambrosini, 2024 | OPEN: 63 (IQR: 60–69) ^b^; RA: 64 (IQR: 60–67) ^b^ | Germany | Single-center | Retrospective cohort | Open-label | Recurrent disease | 85 |
| 18 [37] | Winkens, 2023 | 55, 66, 82, 73, 73, 62 ^c^ | Germany | Single-center | Retrospective cohort | Open-label | Recurrent disease | 6 |
| 19 [18] | Lunger, 2023 | 66 (IQR: 64–69) ^b^ | Germany | Single-center | Retrospective cohort | Open-label | Primary intermediate -/ high-risk | 35 |
| 20 [38] | Knipper, 2021 | 67 (IQR: 63–74) ^b^ | Germany | Multicenter | Retrospective cohort | Open-label | Recurrent disease | 40 |
| 21 [39] | Darr, 2020 | 72 (median) ^b^ | Germany | Single-center | Prospective cohort | Open-label | Primary high-risk | 10 |
| 22 [40] | Heuvel, 2020 | 67, 71, 58, 73, 63^c^ | Netherlands | Single-center | Prospective cohort | Open-label | Primary high-risk | 5 |
| 23 [41] | Heuvel, 2022 | 65.6 (IQR: 60.8–70.5) ^b^ | Netherlands | Single-center | Prospective cohort | Open-label | Primary high-risk | 15 |
| 24 [42] | Darr, 2021 | 66 (IQR: 59–69) ^b^ | Germany | Single-center | Prospective cohort | Open-label | Primary intermediate -/ high-risk | 10 |
| 25 [43] | Muraglia, 2023 | NR | Italy | Single-center | Prospective cohort | Open-label | Primary high-risk | 2 |
| 26 [15] | Darr, 2023 | 65.6 (IQR: 60.8–70.5) ^b^ | Germany | Multicenter | Prospective cohort | Open-label | Primary high-risk | 10 |
| 27 [44] | Moraitis, 2025 | 68 (range: 60–80) ^b^ | Germany | Single-center | Prospective cohort | Open-label | Primary high-risk | 7 |
| 28 [45] | Mazzucato, 2024 | 71 (IQR: 66–72)^b^ | Germany | Multicenter | Retrospective cohort | Open-label | Recurrent disease | 13 |

ᵃ = mean ± SD; ᵇ = median (IQR) or median (range); ᶜ = individual values
