# Supplementary material for: Diagnostic accuracy of PSMA-targeted radioguided surgery in prostate cancer at multiple anatomical levels: a systematic review and meta-analysis
Source: Eur J Nucl Med Mol Imaging. 2026 Mar 27;53(8):4850–61. doi: 10.1007/s00259-026-07773-x (PMC13249658; doi:10.1007/s00259-026-07773-x)
Supplement: Supplementary file 20 — Supplementary file20 (DOCX 74.4 KB) [file 259_2026_7773_MOESM20_ESM.docx]

**Article Title:**

Diagnostic Accuracy of PSMA-Targeted Radioguided Surgery in Prostate Cancer at Multiple Anatomical Levels: A Systematic Review and Meta-analysis

**Journal:**

European Journal of Nuclear Medicine and Molecular Imaging (EJNMMI)

**Authors:**

Fang Wen, Laura Schäfer, Xinlin Zheng, Hao Huang, Walter Noordzij, Matthias Saar, Felix M. Mottaghy, Susanne Lütje

**Corresponding Author:**

Univ.-Prof. Dr. Dr. med. Susanne Lütje

Department of Nuclear Medicine

University Hospital RWTH Aachen

Pauwelsstraße 30

52074 Aachen

Germany

Email: sluetje@ukaachen.de

**File Type:**

Supplementary Material – Supplementary Table S5

**Supplementary Table S5.** Characteristics of Included Studies (Surgical and Radioguided Procedures)

| **Ref** | **Author, year** | **Preoperative imaging**  **agent** | **Preoperative PSA (ng/mL)** | **Type of surgery** | **Gamma Probe Type** | **RGS PSMA agent** | **Tracer Dose (intraoperative)** | **Complication Rate**  **(≥ Clavien Grade III)** | **Complication Type** | **Adjuvant Treatment**  **ration** |
| --- | --- | --- | --- | --- | --- | --- | --- | --- | --- | --- |
| 1 [21] | Collamati, 2020 | [^18^F]DCFPyL | 4.4,5.3,8.3,2.7,6.4,9.3, 4.4^c^ | RA  RP + PLND | β-probe | [^68^Ga]Ga-PSMA-11 | Median 68 MBq (IQR 63.5–82) | NR | NR | NR |
| 2 [22] | Jilg, 2020 | [^68^Ga]Ga-PSMA-11 | 7.9± 12.9^a^ | Open  RP + ePLND sLND | γ-probe(germanium detector used post-operatively) | [^111^In]In-PSMA-617 | Mean 110 ± 14 MBq | 2/23 | Lymphorrhea requiring drainage | 10/23 |
| 3 [23] | Mix,2021 | [^68^Ga]Ga-PSMA-11 /  [^18^F]PSMA (type not reported) | 44.6 ± 45.5^a^ | Open  RP sLND | γ-probe(germanium detector used post-operatively) | [^99m^Tc]Tc-PSMA-I&S | Mean 638 ± 57 MBq | NR | NR | 3/6 |
| 4 [14] | de Barros, 2022 | [^68^Ga]Ga-PSMA-11 / [18F]DCFPyL | Median 1.02 (IQR 0.46–2.43)^b^ | RA sLND | γ-probe | [^99m^Tc]Tc-PSMA-I&S | Mean 548.3 ± 38.5 MBq | 1/19 | Serosal injury to the bladder | NR |
| 5 [24] | Gondoputro, 2022 | [^68^Ga]Ga-PSMA-11 | Median 9.15 (IQR 6.0–21.2) ^b^ | RA  RP + ePLND | γ-probe | [^99m^Tc]Tc-PSMA-I&S | 500 MBq | 0/12 | NA | 6/12 |
| 6 [25] | Knipper, 2023 | [^68^Ga]Ga-PSMA-11 | 1.0 (IQR: 0.5–1.9) ^b^ | Open sLND | γ-probe | [^99m^Tc]Tc-PSMA-I&S | Mean 571 MBq (Range: 221–857) | 24/364 | Lymphocele, Thrombosis, Urinary retention with suprapubic catheterization, Bladder injury, Intestinal perforation,Pelvic hematoma, Persistent lymphorrhea with chyleous ascites, Ureteral stenosis/injury,Wound infection,Sigma perforation with septic shock | 121/358 |
| 7 [26] | Yılmaz, 2022 | [^68^Ga]Ga-PSMA-11 | 40.9 ± 76.2 ^a^ | RA  RP + ePLND | γ-probe | [^99m^Tc]Tc-PSMA-I&S | 630（555–770）MBq | 0/15 | NA | 3/15 |
| 8 [27] | Gandaglia,2022 | [^68^Ga]Ga-PSMA-11 | 8.7 (IQR: 4.8–15.5) ^b^ | RA  RP + ePLND | γ-probe | [^99m^Tc]Tc-PSMA-I&S | Median activity: 735 MBq | 0/12 | NA | NR |
| 9 [28] | Koehler, 2023 | [^68^Ga]Ga-PSMA-I&T | Median 0.74 (IQR 0.41–1.54)^b^ | Open sLND | γ-probe | [^99m^Tc]Tc-MIP-1404 | 745（669–787）MBq | NR | NR | 4/9 |
| 10 [29] | Stibbe, 2023 | NR | 15 (IQR: 9.3–22.0) ^b^ | RA  RP + ePLND | VisionSense NIR fluorescence system | OTL78 (NIR-PSMA) | 0.03 mg/kg | 3/18 | Lymphocele, Haematoma, Haematuria, Haemorrhage, Urosepsis, Wound complication | NR |
| 11 [30] | Falkenbach, 2025 | NR | 1.95 (IQR: 1.36–3.20) ^b^ | Open sLND | γ-probe | [^99m^Tc]Tc-PSMA-I&S | NR | 9/111 , | Ureteral injury, Intestinal injury, Bleeding complications requiring surgical repair, Wound infection | NR |
| 12 [31] | Mayr, 2024 | [^68^Ga]Ga-PSMA-11 / [^18^F]AIF-PSMA-11 | 1.2 (IQR 0.6–3.0) ^b^ | Open sLND | γ-probe | [^99m^Tc]Tc-PSMA-I&S | 497 MBq | 8/50 , | Ureteral injury / ureter-related complications, Fascial dehiscence, Postoperative hematoma | 29/50 |
| 13 [32] | Harke, 2024 | [^99m^Tc]Tc-PSMA-I&S | 11.0 (range: 2.98–17.8) ^b^ | RA  RP + ePLND | γ-probe | [^99m^Tc]Tc-PSMA-I&S | Median 682 MBq (range: 510–734 MBq) | 0/12 | NA | NR |
| 14 [33] | Quarta, 2024 | [^68^Ga]Ga-PSMA-11 / [^18^F]PSMA-1007 | 8.5 (IQR: 4.6–16.0) ^b^ | RA  RP + ePLND | γ-probe | [^99m^Tc]Tc-PSMA-I&S | 734 MBq | NR | NR | NR |
| 15 [34] | Collamati, 2024 | NR | 10 (IQR: 8.4–20) ^b^ | RA  RP + ePLND | β-probe | [^68^Ga]Ga-PSMA-11 | Median 100 MBq (range: 84–107 MBq) | NR | NR | NR |
| 16 [35] | Schilham, 2024 | [^18^F]PSMA (type not reported) | 69 (range: 57–79) ^b^ | RA  RARP + ePLND / ePLND | γ-probe | [^111^In]In-PSMA-I&T | Mean 157 MBq (range: 151.8–164.2 MBq) | 0/16 | NA | 13/20 |
| 17 [36] | Ambrosini, 2024 | NR | OPEN: 0.58 (IQR: 0.38–0.87) ^b^; RA: 0.44 (IQR: 0.31–0.71) ^b^ | RA sLND | γ-probe | [^99m^Tc]Tc-PSMA-I&S | NR | 4/61 | NR | 40/61 |
| 18 [37] | Winkens, 2023 | [^68^Ga]Ga-PSMA-I&T | 5.1, 0.7, 0.5, 3.0, 2.4, 3.3^c^ | Open sLND | γ-probe | [^67^Ga]Ga-PSMA-I&T | Mean 152.3 MBq (sd: 1.27 MBq) | NR | NR | NR |
| 19 [18] | Lunger, 2023 | NR | 18 (IQR: 9–35) ᵇ | Open  RP + ePLND | γ-probe | [^99m^Tc]Tc-PSMA-I&S | Mean 150 MBq (sd: 40 MBq; range: 86–298 MBq) | NR | NR | 25/35 |
| 20 [38] | Knipper, 2021 | NR | 0.9 (IQR: 0.5–1.7) ᵇ | Open sLND | γ-probe | [^111^In]In-PSMA-I&T / [^99m^Tc]Tc-PSMA-I&S | NR | 3/40 | Urethral stricture, rectal injury, ureteral injury | 33/40 |
| 21 [39] | Darr, 2020 | [^68^Ga]Ga-PSMA-11 | 9.04 (median) ᵇ | Open  RP + ePLND | CLI | [^68^Ga]Ga-PSMA-11 | （1.8-2.2 MBq/kg） | NR | NR | NR |
| 22 [40] | Heuvel, 2020 | [^68^Ga]Ga-PSMA-11 | 29.9, 4.4, 5.3, 8.3, 6.4^c^ | RA  RP + ePLND | CLI | [^68^Ga]Ga-PSMA-11 | Mean 83 MBq (sd: 19 MBq) | NR | NR | NR |
| 23 [41] | Heuvel, 2022 | [^68^Ga]Ga-PSMA-11 / [^18^F]DCFPyL | 8.15 (IQR: 5.7–12.9)ᵇ | RA  RP + ePLND | CLI | [^68^Ga]Ga-PSMA-11 | Mean 69 MBq (sd: 27 MBq) | NR | NR | NR |
| 24 [42] | Darr, 2021 | [^68^Ga]Ga-PSMA-11 | 12.0 (IQR: 5.6–16.0) ^b^ | Open  RP + ePLND | CLI | [^68^Ga]Ga-PSMA-11 | Range: 1.8–2.2 MBq | NR | NR | NR |
| 25 [43] | Muraglia, 2023 | [^68^Ga]Ga-PSMA-11 | 10, 8^c^ | RA  RP + ePLND | specimen PET/CT | [^68^Ga]Ga-PSMA-11 | Mean 85.0 MBq (sd: 7.1 MBq) | NR | NR | NR |
| 26 [15] | Darr, 2023 | [^68^Ga]Ga-PSMA-11 / [^18^F]PSMA-1007 | 8.15 (IQR: 5.7–12.9) ^b^ | RA  RP + ePLND | specimen PET/CT | [^68^Ga]Ga-PSMA-11 / [^18^F]PSMA-1007 | NR | NR | NR | NR |
| 27 [44] | Moraitis, 2025 | [^18^F]PSMA-1007 | 8.27 (range: 4.93–19.0)^b^ | RA  RP + ePLND | specimen PET/CT | [^18^F]PSMA-1007 | Mean 3.7 MBq/kg (range: 3.2–4.8 MBq/kg) | NR | NR | 2/7 |
| 28 [45] | Mazzucato, 2024 | NR | 0.93 (IQR: 0.48–1.71)^b^ | RA  ePLND | γ-probe | [^99m^Tc]Tc-PSMA-I&S | NR | 0/13 | NA | NR |

ᵃ = mean ± SD; ᵇ = median (IQR) or median (range); ᶜ = individual values.
