# Supplementary material for: Diagnostic accuracy of PSMA-targeted radioguided surgery in prostate cancer at multiple anatomical levels: a systematic review and meta-analysis
Source: Eur J Nucl Med Mol Imaging. 2026 Mar 27;53(8):4850–61. doi: 10.1007/s00259-026-07773-x (PMC13249658; doi:10.1007/s00259-026-07773-x)
Supplement: Supplementary file 21 — Supplementary file21 (DOCX 75.0 KB) [file 259_2026_7773_MOESM21_ESM.docx]

**Article Title:**

Diagnostic Accuracy of PSMA-Targeted Radioguided Surgery in Prostate Cancer at Multiple Anatomical Levels: A Systematic Review and Meta-analysis

**Journal:**

European Journal of Nuclear Medicine and Molecular Imaging (EJNMMI)

**Authors:**

Fang Wen, Laura Schäfer, Xinlin Zheng, Hao Huang, Walter Noordzij, Matthias Saar, Felix M. Mottaghy, Susanne Lütje

**Corresponding Author:**

Univ.-Prof. Dr. Dr. med. Susanne Lütje

Department of Nuclear Medicine

University Hospital RWTH Aachen

Pauwelsstraße 30

52074 Aachen

Germany

Email: sluetje@ukaachen.de

**File Type:**

Supplementary Material – Supplementary Table S6

**Supplementary Table S6.** Pathological Outcomes and Previous Therapies

| **Ref.** | **Author, year** | **Time from**  **Last therapy to guided surgery** | **Gleason score** | | | | **Previous**  **therapies** | **ISUP** | | | | **Pathological T-stage** | | | | |
| --- | --- | --- | --- | --- | --- | --- | --- | --- | --- | --- | --- | --- | --- | --- | --- | --- |
|  |  |  | **≤7** | **8** | **≥9** | **NA** |  | **1-2**  **(n)** | **3**  **(n)** | **4-5**  **(n)** | **NR**  **(n)** | **≤2**  **(n)** | **3a**  **(n)** | **3b**  **(n)** | **4**  **(n)** | **NR**  **(n)** |
| 1 [21] | Collamati, 2020 | NA | 0 | 4 | 3 |  | NR | 0 | 0 | 7 | 0 | 4 | 1 | 2 | 0 | 0 |
| 2 [22] | Jilg, 2020 | 4.9 ± 3.7y^a^ | 11 | 5 | 7 |  | RP, RT | 4 | 7 | 12 | 0 | NR | NR | NR | NR | NR |
| 3 [23] | Mix,2021 | NR | 2 | 3 | 1 |  | RP, sLND | 0 | 2 | 4 | 0 | NR | NR | NR | NR | NR |
| 4 [14] | de Barros, 2022 | 25.5（IQR 11.3-46.7）mo^b^ | NR | NR | NR |  | RP (±LND), RT | NR | NR | NR | NR | NR | NR | NR | NR | NR |
| 5 [24] | Gondoputro, 2022 | NA | 0 | 3 | 9 |  | None | 0 | 0 | 12 | 0 | 88 | 1 | 3 | 0 | 0 |
| 6 [25] | Knipper, 2023 | 54(IQR 28-93) mo^b^ | 223 | 4 | 60 |  | RP, ePLND | 123 | 127 | 100 | 14 | 145 | 105 | 107 | 0 | 14 |
| 7 [26] | Yılmaz, 2022 | NA | 7 | 1 | 7 |  | None | 3 | 4 | 8 | 0 | NR | NR | NR | NR | NR |
| 8 [27] | Gandaglia,2022 | NA | 6 | 4 | 2 |  | RP, ePLND | 1 | 5 | 6 | 0 | 9 | 0 | 3 | 0 | 0 |
| 9 [28] | Koehler, 2023 | 36(IQR 15-42) mo^b^ | 6 | 0 | 2 |  | RP, RT | 1 | 5 | 3 | 0 | 2 | 3 | 3 | 1 | 0 |
| 10 [29] | Stibbe, 2023 | NR | 15 | 0 | 3 |  | RP | 7 | 8 | 3 | 0 | 17 | 1 | 0 | 0 | 0 |
| 11 [30] | Falkenbach, 2025 | 41(IQR 25-70) mo^b^ | 14 | 1 | 3 |  | RP, RT | NR | NR | NR | NR | NR | NR | NR | NR | NR |
| 12 [31] | Mayr, 2024 | 44(IQR 23-92) mo^b^ | 30 | 7 | 10 |  | RP, RT | 30 | 0 | 17 | 3 | 21 | 14 | 13 | 0 | 2 |
| 13 [32] | Harke, 2024 | NA | 2 | 4 | 6 |  | None | 0 | 2 | 10 | 0 | NR | NR | NR | NR | NR |
| 14 [33] | Quarta, 2024 | NA | 14 | 12 | 4 |  | None | 0 | 8 | 22 | 0 | 9 | 15 | 6 | 0 | 0 |
| 15 [34] | Collamati, 2024 | NA | 0 | 3 | 4 |  | None | NR | NR | NR | NR | 0 | 7 | 0 | 0 | 0 |
| 16 [35] | Schilham, 2024 | NA | NA | NA | NA |  | NA | 6 | 3 | 11 | 0 | 6 | 5 | 8 | 1 | 0 |
| 17 [36] | Ambrosini, 2024 | Open: 42 (IQR 22–74)mo^b^ Robot: 35 (13–76) mo^b^ | 22 | 57 | | 6 | RP, RT | 22 | 57 | | 6 | 30 | 28 | 21 | 0 | 6 |
| 18 [37] | Winkens, 2023 | 5.2 (1.5–8.6) y ^b^ | 4 | 1 | 1 |  | RP, RT | 3 | 1 | 2 | 0 | 4 | 1 | 1 | 0 | 0 |
| 19 [18] | Lunger, 2023 | 36 (IQR 18–42) d^b^ | 14 | 21 | |  | None | 14 | | 21 | 0 | 6 | 5 | 23 | 1 | 0 |
| 20 [38] | Knipper, 2021 | 6.4 (IQR 3.4–9.6)y^b^ | 19 | 6 | 14 |  | RP, RT | NR | NR | NR | NR | 18 | 15 | 7 | 0 | 0 |
| 21 [39] | Darr, 2020 | 25.5 (IQR: 11.3–46.7) mo^b^ | 2 | 4 | 4 |  | RT | 0 | 2 | 8 | 0 | 2 | 5 | 3 | 0 | 0 |
| 22 [40] | Heuvel, 2020 | 6.4 (IQR: 3.4–9.6) yr^b^ | 0 | 3 | 2 |  | RP, RT | 0 | 0 | 5 | 0 | 2 | 2 | 1 | 0 | 0 |
| 23 [41] | Heuvel, 2022 | NR | 11 | 5 | 7 |  | RP | 1 | 2 | 12 | 0 | 4 | 6 | 5 | 0 | 0 |
| 24 [42] | Darr, 2021 | NR | 1 | 2 | 2 |  | NA | 3 | 2 | 5 | 0 | NR | NR | NR | NR | NR |
| 25 [43] | Muraglia, 2023 | NA | 0 | 1 | 1 |  | RP | 0 | 0 | 2 | 0 | 1 | 1 | 0 | 0 | 0 |
| 26 [15] | Darr, 2023 | NA | 2 | 2 | 6 |  | RP | 1 | 2 | 7 | 0 | 2 | 4 | 4 | 0 | 0 |
| 27 [44] | Moraitis, 2025 | NR | 1 | 2 | 4 |  | RP | 1 | 0 | 6 | 0 | 4 | 2 | 1 | 0 | 0 |
| 28 [45] | Mazzucato, 2024 | 49 (IQR 25–70) mo^b^ | NR | NR | NR | NR | RP (±LND), RT | NR | NR | NR | NR | NR | NR | NR | NR | NR |

ᵃ = mean ± SD; ᵇ = median (IQR) or median (range); ᶜ = individual values. Time units: y = years; mo = months; d = days
