# Supplementary material for: Diagnostic accuracy of PSMA-targeted radioguided surgery in prostate cancer at multiple anatomical levels: a systematic review and meta-analysis
Source: Eur J Nucl Med Mol Imaging. 2026 Mar 27;53(8):4850–61. doi: 10.1007/s00259-026-07773-x (PMC13249658; doi:10.1007/s00259-026-07773-x)
Supplement: Supplementary file 22 — Supplementary file22 (DOCX 18 KB) [file 259_2026_7773_MOESM22_ESM.docx]

**Article Title:**

Diagnostic Accuracy of PSMA-Targeted Radioguided Surgery in Prostate Cancer at Multiple Anatomical Levels: A Systematic Review and Meta-analysis

**Journal:**

European Journal of Nuclear Medicine and Molecular Imaging (EJNMMI)

**Authors:**

Fang Wen, Laura Schäfer, Xinlin Zheng, Hao Huang, Walter Noordzij, Matthias Saar, Felix M. Mottaghy, Susanne Lütje

**Corresponding Author:**

Univ.-Prof. Dr. Dr. med. Susanne Lütje

Department of Nuclear Medicine

University Hospital RWTH Aachen

Pauwelsstraße 30

52074 Aachen

Germany

Email: sluetje@ukaachen.de

**File Type:**

Supplementary Material – Supplementary Table S7

**Supplementary Table S7.** Subgroup and Moderator Analysis of Diagnostic Odds Ratios

| Covariate | Subgroup | Studies, n | Pooled Log DOR*  (95% CI) | *P*-value  (vs. Reference) | *P-*value  (Moderator Test) |
| --- | --- | --- | --- | --- | --- |
| Modality |  |  |  |  | < 0.0001* |
|  | pre-operative | 22 | 2.99 (2.08, 3.90) | Reference |  |
|  | RGS_ex vivo | 16 | 5.17 | 0.0019** |  |
|  | RGS_in vivo | 20 | 5.77 | < 0.0001*** |  |
| Analysis_level |  |  |  |  | 0.0001* |
|  | lesion-based | 18 | 4.42 (3.46, 5.37) | Reference |  |
|  | lymph node-based | 9 | 7.08 | 0.0011** |  |
|  | patient-based | 22 | 3.43 | 0.1468 |  |
|  | region-based | 9 | 4.93 | 0.5252 |  |
| Study_design |  |  |  |  | 0.1095 |
|  | prospective | 35 | 4.94 (4.16, 5.71) | Reference |  |
|  | retrospective | 23 | 3.91 | 0.1095 |  |
| PSMA_Agent |  |  |  |  | 0.1654 |
|  | [^99^mTc]PSMA-I&S | 30 | 4.80 (3.99, 5.62) | Reference |  |
|  | [^111^In]PSMA-617 | 2 | 6.99 (4.80, 9.80) | 0.1436 |  |
|  | [^111^In]PSMA-I&T | 2 | 4.02 (0.63, 7.41) | 0.6494 |  |
|  | [^111^In]PSMA-I&T / [^99m^Tc]PSMA-I&S | 1 | 2.79 (-2.13, 7.62) | 0.4233 |  |
|  | [^18^F]PSMA-1007 | 1 | 4.86 (0.25, 9.48) | 0.9787 | 0.1531 |
|  | [^67^Ga]PSMA-I&T | 4 | 2.56 (0.31, 4.80) | 0.1335 |  |
|  | [^68^Ga]PSMA-11 | 12 | 3.46 (2.03, 4.90) | 0.1043 |  |
|  | [^68^Ga]PSMA-11 / [^18^F]PSMA-1007 | 1 | 3.53 (-3.40, 10.47) | 0.6576 |  |
|  | [^99m^Tc]MIP-1404 | 3 | 3.94 (2.51, 5.37) | 0.5471 |  |
|  | OTL78 (NIR-PSMA) | 2 | 7.77 (4.68, 10.85) | 0.0599 |  |
| Fluorescence used |  |  |  |  | 0.0302 |
|  | No | 56 | 4.42 (3.81, 5.04) | Reference |  |
|  | Yes | 2 | 7.76 (6.15, 9.36) | 0.0302 * |  |
| Study center |  |  |  |  | 0.7973 |
|  | Multicenter | 8 | 4.75 (3.01, 6.49) | Reference |  |
|  | Single-center | 50 | 4.51 (2.64, 6.12) | 0.7973 |  |
| Preoperative SPECT |  |  |  |  | 0.6926 |
|  | No | 19 | 4.72 (3.63, 5.82) | Reference |  |
|  | Yes | 39 | 4.45 (3.12, 5.78) | 0.6926 |  |
| Intraoperative probe |  |  |  |  | 0.0129* |
|  | γ-probe | 39 | 4.42 (3.72, 5.12) | Reference |  |
|  | CLI | 5 | 3.00 (0.84, 6.14) | 0.1983 |  |
|  | specimen PET/CT | 3 | 3.91 (0.27, 7.85) | 0.7341 |  |
|  | VisionSense NIR fluorescence system | 2 | 7.74 (4.56, 10.94) | 0.0231* |  |
|  | β-probe | 6 | 3.97 (1.30, 6.94) | 0.6516 |  |
|  | γ-probe (incl. germanium detector)* | 3 | 7.34 (4.03, 10.29) | 0.0111* |  |
| Surgery Type |  |  |  |  | 0.6782 |
|  | RA (reference) | 40 | 4.62 (3.88, 5.36) | Reference |  |
|  | Open | 18 | 4.33 (3.17, 5.50) | 0.6782 |  |

*Pooled log DOR values for non-reference subgroups are presented as point estimates. 95% confidence intervals are available from model output.

*This subgroup includes studies that reported use of γ-probes in combination with germanium detectors, though not all applied intraoperatively.
