# Supplementary material for: Diagnostic accuracy of PSMA-targeted radioguided surgery in prostate cancer at multiple anatomical levels: a systematic review and meta-analysis
Source: Eur J Nucl Med Mol Imaging. 2026 Mar 27;53(8):4850–61. doi: 10.1007/s00259-026-07773-x (PMC13249658; doi:10.1007/s00259-026-07773-x)
Supplement: Supplementary file 23 — Supplementary file23 (DOCX 14 KB) [file 259_2026_7773_MOESM23_ESM.docx]

**Article Title:**

Diagnostic Accuracy of PSMA-Targeted Radioguided Surgery in Prostate Cancer at Multiple Anatomical Levels: A Systematic Review and Meta-analysis

**Journal:**

European Journal of Nuclear Medicine and Molecular Imaging (EJNMMI)

**Authors:**

Fang Wen, Laura Schäfer, Xinlin Zheng, Hao Huang, Walter Noordzij, Matthias Saar, Felix M. Mottaghy, Susanne Lütje

**Corresponding Author:**

Univ.-Prof. Dr. Dr. med. Susanne Lütje

Department of Nuclear Medicine

University Hospital RWTH Aachen

Pauwelsstraße 30

52074 Aachen

Germany

Email: sluetje@ukaachen.de

**File Type:**

Supplementary Material – Supplementary Table S8

**Supplementary Table S8.** Meta-regression Model for Independent Predictors of Diagnostic Performance

| Covariate | Subgroup / Category | Estimate (95% CI) | *P*-value |
| --- | --- | --- | --- |
| Modality | Pre-operative | Reference |  |
|  | RGS_ex vivo | 1.59 (0.14, 3.04) | 0.0313* |
|  | RGS_in vivo | 2.37 (1.21, 3.53) | <0.0001*** |
| Analysis level | Lesion-based | Reference |  |
|  | Lymph node-based | 1.69 (0.23, 3.16) | 0.0235* |
|  | Patient-based | –0.59 (–1.85, 0.67) | 0.3576 |
|  | Region-based | 0.04 (–1.45, 1.54) | 0.9543 |
| Fluorescence used | No | Reference |  |
|  | Yes | 1.83 (–0.51, 4.17) | 0.1246 |
| Intraoperative probe | γ-probe | Reference |  |
|  | CLI | –0.90 (–3.04, 1.23) | 0.4068 |
|  | Specimen PET/CT | –1.12 (–3.93, 1.69) | 0.4362 |
|  | β-probe | 0.28 (–1.40, 1.96) | 0.7432 |
|  | γ-probe (incl. germanium detector)* | 2.61 (0.56, 4.66) | 0.0126* |

*This subgroup includes studies that reported use of γ-probes in combination with germanium detectors, though not all applied intraoperatively.
